# Beilstein Journal of Organic Chemistry

**DOI:** 10.1186/1860-5397-1-1

**Published:** 2005-08-26

**Authors:** Jonathan Clayden

**Affiliations:** 1 Editor - Beilstein Journal of Organic Chemistry, Department of Chemistry, University of Manchester, Oxford Road, Manchester M13 9PL, UK

The creative science of Organic Chemistry is one of the few which has the power constantly to rejuvenate itself, because almost uniquely it studies what it also creates. The flourishing of organic chemistry during the 20^th^ century has led, at the beginning of the 21^st^ century, to a realisation that we are only just starting to etch the surface of what can be achieved with the chemical tools now in our hands and with those still to be discovered. Four years ago, the Nobel prize in chemistry celebrated chemists' achievements in asymmetric catalysis – and even since then the face of catalysis is changing, with for example metathesis and organocatalytic methods constantly breaking into new areas. Synthesis is changing too – it is arguably now a matter of when, rather than whether, a complex new compound can be made, and this new found confidence means that the choice of target is more important than ever. Chemical genetics is standing up to this challenge – which molecules do we make out of the billions we could make? In the past nature told us, and to some extent she still holds our hands (almost literally where asymmetry is concerned) while we choose. But not only are organic molecules being made to interact with biological systems, but whole systems of supramolecular complexity are being built from scratch: para-biologies built, just like the original kind, out of organic chemistry. The synthesis of molecular motors, or sensors, of springs and levers makes possible this new molecular engineering at the nanometre scale for eventual uses which only the future will tell.

Expanding scientific horizons are being matched by expanding geographical horizons: research is global, and many countries, particularly in Asia, are investing in the development of chemistry as never before. It is within this environment of expanding scientific horizons that we are now launching, and presenting to you, the *Beilstein Journal of Organic Chemistry*. Unlike almost all other journals in chemistry, the *Beilstein Journal of Organic Chemistry* is an open access journal – all issues are fully and permanently available online to all, there is no subscription charge, and the copyright remains with the authors of the articles.

An historic mission of the Beilstein-Institut has been to improve the quality of information in organic chemistry. Consistent with this mission it is the aim of the Institute to publish a high quality journal that will in due course stand comparison with the best in chemistry. The *Beilstein Journal of Organic Chemistry* must be one in which authors are proud to publish their work, and not only because it is open access. We are committed to excellence in refereeing through the use of rigorous peer review, and our broad and experienced Editorial Advisory Board will assist us in maintaining the quality of the journal's output.

The advent of internet-based online publishing provides a great opportunity to restore power to the researcher, both as an author and as a reader. As authors you are able to see your work published more quickly and disseminated more widely; you are also in a stronger position to protect your intellectual property rights than in a print world where access was controlled by the publisher. As readers you have potentially greater access to more research information than ever before. The open access model is already well established in the life sciences and the Beilstein-Institut is pleased to offer a journal to serve research chemists in the same way.

As online journals develop it is inevitable that access models will evolve with them. The open access model adopted by the Beilstein-Institut may not be applicable to all, but it will offer effective refereeing, rapid publication, worldwide dissemination and permanent archiving: and it does so while allowing the author to retain copyright. To be successful in the long-term other access models will probably have to do likewise. What form they will take only the future can tell. What we can say now is that the time when access to scientific information was granted only to those who could afford high subscription prices, and where authors had to surrender their intellectual property rights, is drawing to a close.

Online publishing offers many further advantages beyond the rapidity offered (consistent with the maintenance of quality) by electronic handling of manuscripts right up to the point of publication. Online versions of most other journals are still bound by the need for consistency with a printed equivalent. But for the purely online Beilstein Journal of Organic Chemistry, colour, video files, and hyperlinking pose no problems. Additional information such as experimental or spectroscopic data assisting the reader can, and will, be provided liberally. *Beilstein Journal of Organic Chemistry* will therefore publish both short and long papers accompanied by appropriate experimental data as standard, allowing flexibility in style that was never possible under the now-outdated regime of "communications" and "full papers".

An unfortunate disadvantage of online journals is the loss of that age-old habit of page-flicking – glancing through an issue for something that catches the eye. We have therefore introduced, in an attempt to recapture the value of old-style browsing, the "album" facility: a collection of representative structures or images from a paper, more extensive and less prescriptive than the graphical abstract, which can be scrolled through much as crisp pages of white paper would be flicked. Further unique features, such as the ability to add refereed corrections and comments to published papers, will appear soon.

The *Beilstein Journal of Organic Chemistry*'s pedigree, as a product of the Beilstein-Institut, will ensure its durability and quality, but it also stands as a vibrant new journal in a rejuvenating science. We look forward to your free and frequent participation in this exciting project as readers and authors.

Jonathan Clayden

Editor in Chief

